# Video-fluoroscopic swallowing study scale for predicting aspiration pneumonia in Parkinson’s disease

**DOI:** 10.1371/journal.pone.0197608

**Published:** 2018-06-06

**Authors:** Satoshi Tomita, Tomoko Oeda, Atsushi Umemura, Masayuki Kohsaka, Kwiyoung Park, Kenji Yamamoto, Hiroshi Sugiyama, Hideyuki Sawada

**Affiliations:** Clinical Research Center and Department of Neurology, Utano National Hospital, Kyoto, Japan; Universita degli Studi di Padova, ITALY

## Abstract

**Introduction:**

A number of video-fluoroscopic swallowing study (VFSS) abnormalities have been reported in patients with Parkinson’s disease (PD). However, the most crucial finding of subsequent aspiration pneumonia has not been validated fully. We conducted a retrospective and case-control study to determine the clinically significant VFSS findings in this population, and to propose a practical scale for predicting aspiration pneumonia in patients with PD.

**Methods:**

We enrolled 184 PD patients who underwent VFSS because of suspected dysphagia. The patients who developed aspiration pneumonia within six months of the VFSS were assigned as cases and the patients without aspiration pneumonia at six months were designated as controls. Logistic regression analysis was performed to determine the prognostic VFSS features based on the data of swallowing 3 mL of jelly, which were used to make a PD VFSS scale (PDVFS). The validity of the new PDVFS was evaluated by ROC analysis. Additionally, we used the survival time analysis to compare time to death between groups, stratified by the PDVFS score.

**Results:**

Twenty-five patients developed aspiration pneumonia. Among the previously-proposed VFSS features, mastication, lingual motility prior to transfer, aspiration, and total swallow time were identified as significant prognostic factors. We combined these factors to form the PDVFS. The PDVFS score ranges from 0 to 12, with 12 being the worst. ROC analysis revealed 92% sensitivity and 82% specificity at a cutoff point of 3. The higher PDVFS group showed shorter time-to-death than the lower PDVFS group (log rank P = 0.001).

**Conclusion:**

Our newly developed VFSS severity scale (based on jelly swallowing) for patients with PD was easy to rate and could predict subsequent aspiration pneumonia and poor prognosis in patients with PD.

## Introduction

Dysphagia is a common complication of the middle and later stages of Parkinson’s disease (PD), occurring in over 80% of patients [1]. The most common cause of death in such patients is aspiration pneumonia, resulting from a preexisting dysphagia [2]. To prevent the serious consequences of dysphagia in PD, the predictive factors of pneumonia development need to be identified.

Video-fluoroscopic swallowing study (VFSS) is a widely performed procedure used for detecting dysphagia and reveals some abnormalities in 75% to 97% of individuals with PD [3–6]. In particular, a VFSS can reveal oropharyngeal dysphagia [7]. In the oral phase, abnormal bolus formation, residue on the tongue, and piecemeal deglutition can be noted. In the pharyngeal phase, pharyngeal dysmotility, pharyngeal stasis, and vallecular residue after swallowing are sometimes observed [4, 8, 9]. However, the factors that are the most crucial for predicting subsequent development of aspiration pneumonia (and its consequent effects on prognosis), among the various abnormal features that may be observed, are not validated fully for patients with PD.

The purpose of the study was to identify the most significant features on VFSS for predicting the development of aspiration pneumonia and poor prognosis in patients with PD and to create a simple VFSS scale that is suitable for practical use.

## Materials and methods

### Study design

To identify the VFSS findings that were associated with development of aspiration pneumonia in patients with PD, we performed a retrospective case-control study. The primary outcome measure was the development of aspiration pneumonia. The patients were divided into two groups: those who developed aspiration pneumonia within six months after VFSS (cases) and those who did not develop aspiration pneumonia (controls). The association between VFSS parameters and pneumonia was analyzed to identify the predictive features by multivariate analysis.

### Subjects

For screening, we recruited consecutive PD patients who had never been diagnosed with aspiration pneumonia, and who underwent VFSS at the Utano National Hospital Parkinson’s Disease Center between July 2005 and July 2015. At our hospital, the typical reasons for VFSS referral were oropharyngeal dysphagia suspected by patients or patients’ caregivers, or objective swallowing problems (e.g., frequent productive cough, sialorrhea at a moderate level or higher, prolonged mealtime duration, or wet voice suggesting penetration or aspiration).

The diagnosis of PD was made according to the UK Brain Bank Clinical Diagnostic Criteria (Steps 1 and 2). All the subjects underwent brain magnetic resonance imaging to exclude other neurologic disorders. The patients were excluded if they were receiving tube feedings or had a tracheostomy; if they had other diseases that could cause dysphagia (e.g., stroke, esophageal cancer, drug-induced encephalopathy, and other neurodegenerative diseases); and if they were observed for fewer than six months after VFSS.

### Definition of aspiration pneumonia

The patients who were newly diagnosed by the supervising physician as demonstrating aspiration pneumonia fulfilled two or more of the following criteria: (1) clinical signs and symptoms compatible with pneumonia (e.g., rales or rhonchi on chest auscultation); (2) changes in inflammatory markers, including white blood cell count >10,000/μL, proportion of neutrophils >80%, or serum C-reactive protein level >60 mg/L; and (3) chest radiography findings of new infiltrates or consolidation in the lower dorsal (gravity dependent) segments, supporting a diagnosis of aspiration pneumonia. The most commonly-observed chest CT finding of a bronchopneumonia pattern with a gravity-dependent distribution [10] provides valuable information for diagnosing aspiration pneumonia [11].

### Data collection

In addition to the VFSS findings, we collected the following clinical data on the day of the VFSS: age, disease duration, unified Parkinson’s disease rating scale part III motor score (UPDRS-3), Hoehn-Yahr stage, dietary intake interventions related to dysphagia (ordinary diet or processed diet), and body mass index (BMI). If available within three months of VFSS, we recorded Mini-mental State Examination (MMSE) scores for cognitive evaluation and serum albumin concentrations.

According to the diagnostic criteria, a clinical diagnosis of aspiration pneumonia was confirmed by two of three investigators (S.T., T.O., and H.S.) by reviewing radiological evidence and checking patients’ clinical signs and laboratory data within the medical record. If a definitive diagnosis was not documented, the diagnosis was made according to discussion amongst the researchers. The diagnosis was double marked by two auditors (H.O. and T.I.) who were physicians with considerable experience treating patients with pneumonia. S.T., T.O., and A.U. obtained other data from medical records, as necessary. All the obtained data were audited and certified as identical to the original medical record by K.W., the clinical research coordinator of our institute and an experienced auditor of clinical research studies.

The author (S.T.) retrospectively evaluated all the prerecorded VFSS movies retrospectively sequences, without the associated clinical data, in strict accordance with the standardized VFSS rating protocol of rating VFSS used in this study.

### VFSS protocol

Registered dietitians prepared a jelly containing a nonionic contrast agent, according to a prescribed recipe. The patients were given 3 mL of jelly with a spoon and were asked to swallow voluntarily. Lateral views of the patients swallowing were recorded using a high-speed (30 frames per second) VFSS system. The VFSS was scored based on digitally recorded data. Anti-Parkinsonism medications were taken within one to two hours before the VFSS to evaluate swallowing function during the “on” medication period.

### Selection of VFSS parameters as predictor variables

We extracted the VFSS parameters that predicted development of aspiration pneumonia in patients with PD from the available literature. A PubMed search was completed for articles published from 1986 to 2015 using the keywords ‘‘Parkinson’s disease”, ‘‘dysphagia”, and ‘‘videofluoroscopy”. Studies that described prominent VFSS features in patients with PD who exhibited dysphagia were selected. We then searched the individual articles for definitions of selected VFSS parameters which were compiled and used as a tool to guide the VFSS rating process. Each variable of the various VFSS parameters was converted to a binary code for further analyses. Continuous variables were transformed to dichotomous based on the most discriminating cutoff point for predicting aspiration pneumonia, as determined by the receiver operating characteristic (ROC) curve.

### Variable selection for Parkinson’s disease VFSS scale

First, we assessed the association between each VFSS parameter and the development of aspiration pneumonia using Fisher’s exact test; parameters with statistically significant (p <0.05) associations and large effects (odds ratio >10.0) were selected for further analysis. Second, using binary logistic regression analysis with stepwise variable selection, we identified the statistically significant parameters to obtain the most suitable model for predicting aspiration pneumonia.

A Parkinson’s disease VFSS scale (PDVFS) was devised based on the total value of the selected variables, which were weighed according to the regression coefficient in the model. The ROC curve of PDVFS for the development of pneumonia was used to test for validity and to determine the reference value (cutoff point) that showed optimal sensitivity and specificity. Additionally, ROC curves of PDVFS stratified by mean age, cognitive impairment (MMSE >24 vs. ≤24), disease severity (Hoehn-Yahr stage 1–3 or 4–5), and dietary interventions (processed or ordinary diet) were plotted to confirm the reproducibility of PDVFS across various conditions.

### Reliability tests for Parkinson’s disease VFSS scale

To examine the inter-rater reliability of the PDVFS, six speech-language-pathologists (SLPs) in our institute with extensive experience treating patients with PD and rating the VFSS sequences assessed the PDVFS sequences according to a standardized protocol. All the SLPs were blinded to the clinical data associated with the patients. The inter-rater reliability was determined as the intraclass correlation coefficient (ICC), based on repeat scoring of 15% of the VFSS (selected randomly), as derived from a two-way random effects analysis of variance model.

For checking the intra-rater reliability of the PDVFS the author re-rated the VFSS more than a month after the first evaluation. The intra-rater reliability was assessed using Cohen’s kappa coefficient.

To evaluate the validity of the PDVFS discriminative properties to predict development of aspiration pneumonia after the VFSS sensitivity analyses in subgroups after stratification by age, cognitive impairment, disease severity, and dietary intervention were performed.

### Survival time analysis of life prognosis

To validate the clinical efficacy of PDVFS, we compared the life prognosis after VFSS among the study participants. All-cause mortality was analyzed and a Kaplan-Meier curve was obtained. Observation was from the time of the VFSS to the day of death or censored; lost to follow-up was regarded as alternative outcome. The statistical significance of the differences was examined by a log rank test.

All the statistical analyzes were performed using PASW Statistics version 18 (SPSS Inc., Chicago, IL, USA). P < 0.05 was considered statistically significant.

### Ethics

This study was approved by the Bioethics Committee of Utano National Hospital (registry number: 28–15) and the protocol was consistent with the principles of the Declaration of Helsinki. All the participants signed an informed consent form prior to enrollment.

## Results

### Subjects for analysis

**Fig 1:**
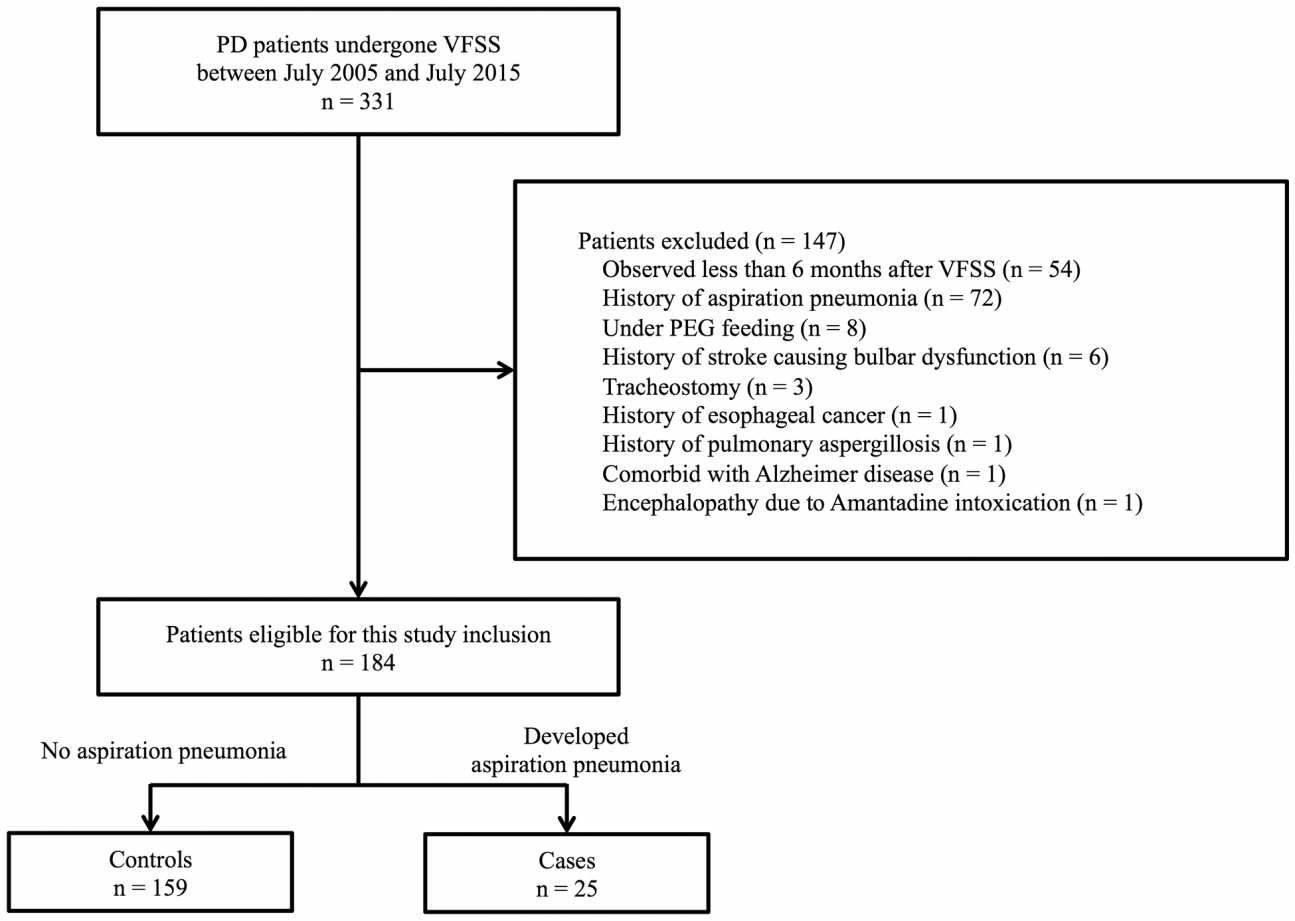
Flow diagram of participants included in the study.

A total of 331 consecutive PD patients who underwent VFSS were recruited for screening. After 147 patients were excluded according to the exclusion criteria, 184 patients were enrolled into the study. Twenty-five patients (13.6%) who developed aspiration pneumonia were assigned as cases, and 159 patients (86.4%) who did not develop aspiration pneumonia were assigned as controls (Fig 1).

### Demographic characteristics of the study population

In the enrolled subjects, the mean (SD) age was 73.0 (8.0) years and the mean (SD) disease duration was 8.6 (5.6) years. The demographic characteristics of the cases and controls are shown in Table 1. In comparison with the controls, the cases were significantly older (P = 0.009) and the proportion of males was higher (P = 0.002). There was no difference in disease duration between the two groups. Compared with the controls, the cases had significantly higher Hoehn-Yahr stages (P = 0.001) and UPDRS-3 scores (P < 0.001), and significantly lower MMSE scores (P < 0.001), BMI (P = 0.004), and serum albumin concentrations (P < 0.001). There were significantly more cases than controls who were placed on processed diets (P = 0.002).

**Table 1:** Demographic and clinical profiles of cases and controls.

	Control	Case	P value
N	159	25	
Age at a VFSS^1^, year (mean ± SD)	72.4 ± 8.1	76.8 ± 7.4	0.009^5^
Male, %	39.0	72.0	0.002^6^
Disease duration, year (mean ± SD)	8.4 ± 5.5	10.0 ± 6.1	0.2^5^
UPDRS-3^2^ score (mean ± SD)	26.8 ± 11.1	38.0 ± 10.9	<0.001^5^
Hoehn-Yahr stage 4–5, %	28.9	64.0	0.001^6^
MMSE^3^ ≤24, %	34.9	86.4	<0.001^6^
BMI^4^ (mean ± SD)	20.9 ± 3.5	19.3 ± 3.4	0.04^5^
Serum albumin, g/dl (mean ± SD)	3.7 ± 0.4	3.4 ± 0.4	<0.001^5^
Patients consuming processed diets, %	39.0	72.0	0.002^6^

^1^ Video-fluoroscopic Swallowing Study

^2^ the unified Parkinson’s disease rating scale part III motor score

^3^ Mini Mental State Examination, missing values in 16 patients (13 in control and 3 in case)

^4^ Body Mass Index

^5^ P value from Mann–Whitney test

^6^ P value from Fisher’s exact test

### VFSS parameters associated with aspiration pneumonia development

PubMed search for articles on VFSS features in patients with PD generated 24 studies (S1 Table). We extracted 26 abnormal parameters from among all VFSS parameters proposed in these studies. There were 13 parameters involving the oral phase: pre-swallow anterior spill, lingual pumping, poor velopharyngeal closure, swallow hesitancy, piecemeal deglutition, lip closure, mastication, lingual motility prior to transfer, bolus formation, premature bolus loss, palatal elevation, poor bolus propulsion, and residue in the oral cavity. There were nine parameters involving the pharyngeal phase: triggering of pharyngeal swallow, vallecular residue, laryngeal elevation, pyriform sinus residue, reduced epiglottal tilt, coating of pharyngeal wall, repeated swallowing, aspiration, and cricopharyngeal dysfunction. Finally, there were four timed parameters: oral transit time, pharyngeal transit time, pharyngeal delay time, and total swallow time.

The continuous data from the parameters of lingual pumping, oral transit time, pharyngeal transit time, pharyngeal delay time and total swallowing time were transformed to dichotomous variables using ROC curves (S1 Fig). The optimal cutoff points were 4 times (lingual pumping), 5 seconds (oral mastication time), 5 seconds (oral transit time), 4 seconds (pharyngeal transit time), and 10 seconds (total swallow time). The definitions of the binary coded values for all parameters are shown in S2 Table.

The odds ratios of the VFSS parameters in predicting aspiration pneumonia based on univariate analyzes are shown in Table 2; 21 of 26 parameters were associated with aspiration pneumonia. Eight of the 21 parameters were incorporated into the logistic regression model; these included mastication, lingual motility prior to transfer, bolus formation, poor bolus propulsion, vallecular residue, repeated swallowing, aspiration, and total swallow time; all parameters were dichotomous. Logistic regression analysis with stepwise variable selection identified mastication, lingual motility prior to transfer, aspiration, and total swallow time as significant predictors of aspiration pneumonia (Table 3). These four parameters were adopted to form the PDVFS.

**Table 2:** Crude odds ratio of each VFSS parameter for the development of aspiration pneumonia.

Parameter	Positive in control, n (%)	Positive in case, n (%)	Odds Ratio	95% CI (lower limit)	95% CI (upper limit)	P value
**Oral phase**						
Pre-swallow anterior spill	1 (1)	0 (0)	0.99	0.98	1.01	1.00
Lingual pumping	54 (34)	9 (36)	0.25	0.03	1.90	0.21
Poor velopharyngeal closure	23 (14)	1 (4)	0.25	0.03	1.90	0.21
Swallow hesitancy	27 (17)	11 (44)	3.84	1.58	9.37	0.006
Piecemeal deglutition	52 (33)	14 (56)	2.62	1.11	6.17	0.04
Lip closure	1 (1)	1 (4)	6.58	0.40	108.79	0.25
Mastication	4 (3)	18 (72)	99.64	26.57	373.70	<0.001
Lingual motility prior to transfer	5 (3)	13 (52)	33.37	10.18	109.35	<0.001
Bolus formation	10 (6)	17 (68)	31.66	11.01	91.07	<0.001
Premature bolus loss	8 (5)	4 (16)	3.60	0.996	12.98	0.06
Palatal elevation	7 (17)	15 (60)	7.33	2.98	18.05	<0.001
Poor bolus propulsion	5 (3)	12 (48)	28.43	8.68	93.17	<0.001
Residue in the oral cavity	95 (60)	22 (88)	4.94	1.42	17.20	0.007
**Pharyngeal phase**						
Triggering of pharyngeal swallow	0 (0)	2 (8)	1.09	0.97	1.22	0.02
Vallecular residue	7 (4)	13 (52)	23.52	7.90	70.02	<0.001
Laryngeal elevation	38 (24)	18 (72)	8.19	3.18	21.09	<0.001
Pyriform sinus residue	24 (15)	15 (60)	8.44	3.40	20.97	<0.001
Reduced epiglottal tilt	12 (8)	5 (20)	5.43	1.57	18.74	0.01
Coating of pharyngeal wall	12 (8)	10 (40)	8.17	3.03	22.05	<0.001
Repeated swallowing	19 (12)	15 (60)	11.05	4.35	28.09	<0.001
Aspiration	2 (1)	4 (16)	14.95	2.58	86.69	0.003
Cricopharyngeal dysfunction	8 (5)	5 (20)	4.72	1.41	15.84	0.02
**Timed parameters**						
Oral transit time	15 (9)	7 (28)	3.73	1.34	10.38	0.02
Pharyngeal transit time	14 (9)	9 (36)	5.83	2.18	15.58	0.001
Pharyngeal delay time	17 (11)	9 (36)	4.70	1.80	12.26	0.003
Total swallow time	24 (15)	18 (72)	14.46	5.46	38.35	<0.001

**Table 3:** Multiple logistic regression model.

	Definitions	Coded value	Coefficient	Odds Ratio	95% CI (lower limit)	95% CI (upper limit)	P value
Mastication	Mastication is slow, hesitant, and delayed with ineffectual movements	0: intact, 1: inadequate	3.7	38.7	7.2	206.6	<0.001
Lingual motility prior to transfer	Tongue movement assisting mastication and bolus formation	0: intact, 1: inadequate	2.1	8.7	1.5	50.5	0.016
Aspiration	Entry of bolus into the lower respiratory tract	0: absent, 1: present	2.6	13.9	1.4	139.5	0.025
Total swallow time	From the initiation of mastication until the tail of the bolus passed through the upper esophageal sphincter	0: ≤10 sec, 1: >10 sec	2.6	14.1	2.9	67.2	0.001

### PDVFS

According to the regression coefficient of the model, PDVFS was calculated as follows: PDVFS = 3.7 × mastication + 2.1 × lingual motility prior to transfer + 2.6 × aspiration + 2.6 × total swallow time (Table 3). To make a simple and practical scale, we rounded off the coefficients by a decimal point to finally obtain the PDVFS (PDVFS = 4 × mastication + 2 × lingual motility prior to transfer + 3 × aspiration + 3 × total swallow time) (Table 4).

**Table 4:** PDVFS.

Parameter	Definitions	Coded value	Score
Mastication	Mastication is slow, hesitant, and delayed with ineffectual movements	Intact	0
		Inadequate	4
Lingual motility prior to transfer	Tongue movement assisting mastication and bolus formation	Intact	0
		Inadequate	2
Aspiration	Entry of bolus into the lower respiratory tract	Absent	0
		Present	3
Total swallow time	From the initiation of mastication until the tail of the bolus passed through the upper esophageal sphincter	≤10 sec.	0
		>10 sec.	3
Total			12

**Fig 2:**
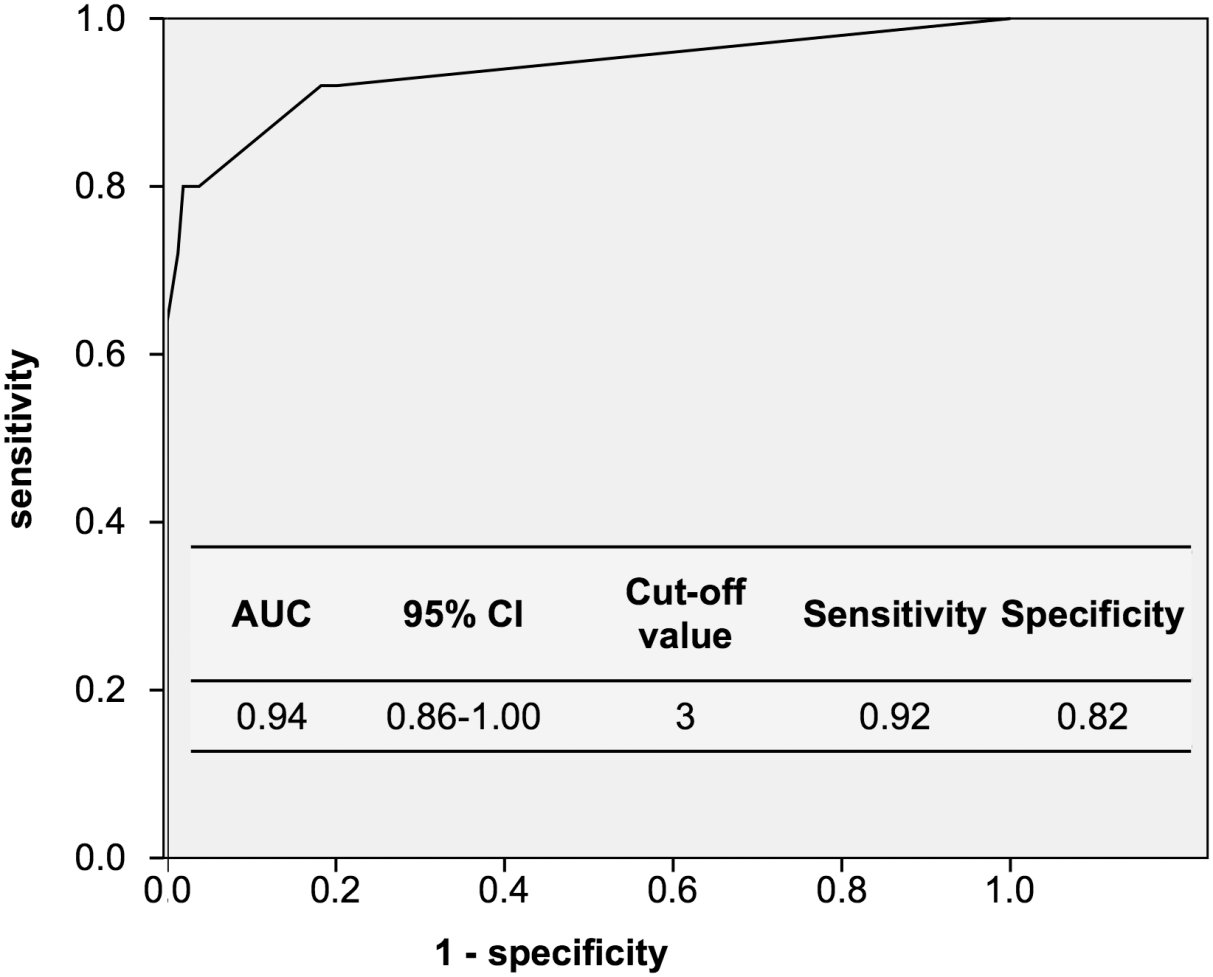
ROC curve of PDVFS and cutoff point for prediction of the development of aspiration pneumonia.

Area under the ROC curve for PDVFS was 0.94 (95% confidence interval: 0.86–1.00). At a cutoff value of 3, the optimal sensitivity and specificity were 0.92 and 0.82, respectively (Fig 2). This diagnostic accuracy of PDVFS was equivalent to that of the original model before rounding-off.

### Reliability tests for PDVFS

Based on the assessments of the recorded VFSS images of 28 study participants (15%) by six SLPs, we found that the inter-rater reliability varied widely among the four components of the PDVFS; however, the total PDVFS score had a moderately acceptable inter-rater reliability (Table 5).

**Table 5:** Inter-rater reliability of PDVFS and composite parameters.

Parameter	Intraclass coefficient (ICC)	95% CI (lower limit)	95% CI (upper limit)
Mastication	0.35	0.21	0.53
Lingual motility prior to transfer	0.52	0.37	0.69
Aspiration	1.00	-	-
Total swallow time	0.97	0.94	0.98
PDVFS Total	0.77	0.62	0.85

The intra-rater reliability of the PDVFS showed moderate (kappa coefficient, 0.82). Subgroup analyzes showed that the PDVFS retained its discriminative properties for predicting development of aspiration pneumonia after the VFSS even after stratification by age, cognitive impairment, disease severity, and dietary intervention (S2 Fig).

### Analysis of prognosis

**Fig 3:**
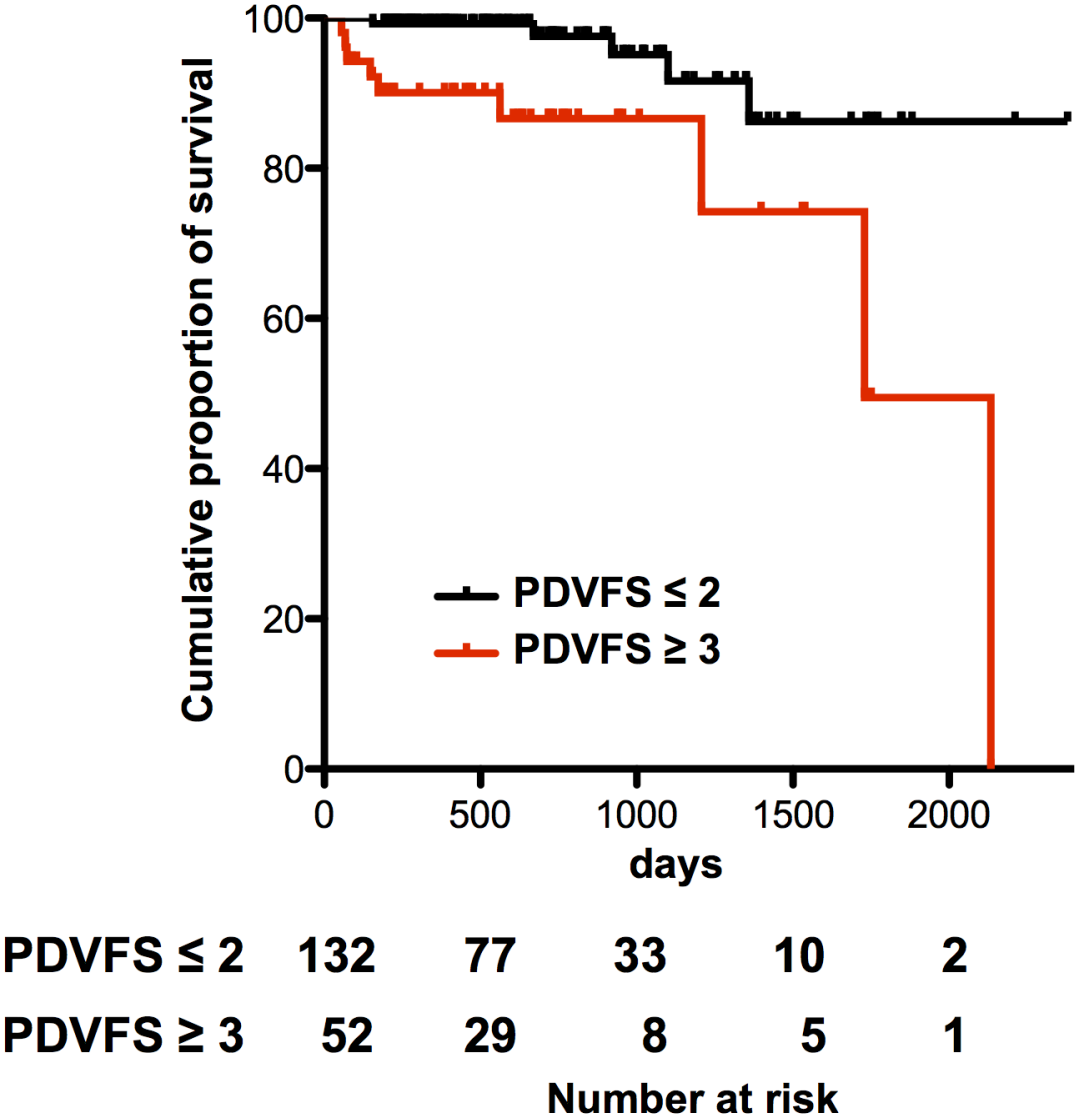
Cumulative survival rates according to PDVFS score (≤2 *vs*.≥3). There was statistically significant difference in the survival rate (Log rank P = 0.001).

Of the 25 cases, 11 patients (44%) discontinued oral feeding immediately after aspiration pneumonia onset and 9 patients (36%) died within the entire observation period because of pneumonia (n = 6), cancer (n = 2), and renal failure (n = 1). Of the 159 controls, 9 patients (6%) discontinued oral feeding and 5 (3%) died because of cancer (n = 2), pneumonia (n = 2), and unknown cause (n = 1). Among the eight patients who died of PD-related complications, the only cause of death was aspiration pneumonia, which was a complication of dysphagia. The mean survival time after pneumonia onset was 364 days (range, 4–1659 days). Fig 3 shows the Kaplan-Meier curve of the cumulative percentage of survival stratified by the PDVFS cutoff score. A PDVFS score of ≥3 points was a significant predictor of poor prognosis (log rank P = 0.001).

## Discussion

Among a series of previously-proposed VFSS features, four parameters (mastication, lingual motility prior to transfer, aspiration, and total swallow time) were strongly associated with the development of aspiration pneumonia in patients with PD. The PDVFS based on the data of swallowing 3 mL of jelly that was proposed in the current study could predict subsequent aspiration pneumonia, regardless of age, disease severity, or cognitive function. Furthermore, the PDVFS could predict life prognosis of patients with PD. The PDVFS was easy to rate and could be a useful index for preventing serious complications of dysphagia in patients with PD.

Among the various VFSS scales that have been developed, the PDVFS is the first scale solely designed for the prediction of subsequent pneumonia development in patients with PD. Han TR et al. [12] examined a sophisticated VFSS scale for pneumonia prediction in patients with stroke which consisted of 14 VFSS features that widely covered the oral to pharyngeal swallowing phases. In comparison of their results and the current results, pharyngeal phase parameters including pharyngeal residue and aspiration were significant features either in PD or stroke patients. In contrast, oral phase parameters including mastication and bolus formation were higher associated with aspiration pneumonia development in PD than in stroke patients. Additionally, there were a remarkable difference in cutoff values of timed parameters between the study by Han et al. and the present study; longer oral and pharyngeal transit times in patients with PD (5.0 sec.) than patients with stroke (1.0–1.5 sec.). These data may indicate that swallowing dysfunctions in the oral phase and a slow bolus transit were important in patients in PD compared to stroke.

Lee JH, et al. [13] and Argolo N, et al. [14] examined VFSS parameters in patients with PD. In their studies, pharyngeal residue, pharyngeal transit time, or piecemeal deglutition are specific predictors of aspiration in PD, and also in our study, all the parameters of pharyngeal phase, including these three parameters, were significantly associated with the development of aspiration pneumonia (Table 2). After adjusting for potential confounders by a logistic regression analysis, a combination of four parameters emerged as significantly associated with aspiration pneumonia: mastication, lingual motility prior to transfer, aspiration, and total swallow time.

The pathophysiology of dysphagia in PD remains unknown. Hypokinesia and bradykinesia due to Parkinsonism can cause motor dysfunction of the tongue and impaired mastication [8, 15]. Therefore, oral transit time is frequently prolonged [16]. In the pharyngeal phase, slow transit and pooling in the valleculae and pyriform sinuses may delay the swallowing reflex [17]. Aspiration and prolonged swallowing time are significant risk factors for aspiration pneumonia [18]. Moreover, silent aspiration is one of the most common findings and an important predictive factor for aspiration in patients with PD [19]. In this study, of the six patients with aspiration, five patients (83%) showed no cough response after aspiration and three patients (50%) developed aspiration pneumonia within six months after VFSS. These three patients were forced to give up oral feeding after recovering from pneumonia. Decreased pharyngeal sensory inputs associated with sensory nerve denervation [20] may contribute to delayed swallowing response and aspiration. On the other hand, a patient who showed a cough response after aspiration also developed pneumonia. Multiple risk factors including swallowing function, decreased cough reflex (leading to silent aspiration), poor oral hygiene, impaired immunity, and reduced mucociliary transport are associated with the development of aspiration pneumonia [10].

The previous literature demonstrated subjective review of a VFSS was associated with insufficient inter- and intra-rater reliability [21]. The parameter of aspiration is well defined and showed high reliability; however, other parameters of oropharyngeal swallow, especially functional components, showed low reliability [22, 23]. Our data were obtained by having each patient swallow once or twice with a predefined amount of a predefined consistency and all the swallows were evaluated according to a strict protocol using binary scales. These modifications were thought to raise inter-rater reliability [22]. The reliability of composite parameters obtained in this study, as with the prior studies, was insufficient. Swallowing is a highly complex functional process and we usually perform VFSS evaluation with full access to a patient’s clinical information and assess the study results using descriptive summaries which allow for, and capture, a wide degree of variability [22]. Taking the difficulty of VFSS evaluation into consideration, we may have to perform a multilateral analysis and a comprehensive evaluation for VFSS study assessments.

In recent studies, age, male sex, dementia, psychosis, postural instability, and early dysphagia were independent predictors of poor prognosis [24–26]. Although pharyngeal dysmotility had been observed in the early stages of dysphagia, patients with PD who were still ambulatory did not frequently develop aspiration pneumonia [27–29]. In this study, sex, age, disease severity, and dementia may have contributed to the development of aspiration pneumonia; however, disease duration did not.

Many PD patients with dysphagia exhibit concomitant reductions in QOL [30] due to insufficient medication intake, malnutrition, and dehydration; these factors may predispose a patient to aspiration pneumonia and subsequent mortality. At the baseline, the cases in this study had already lost weight and most of them were consuming a processed diet, as compared to the controls. In this context dysphagia might have exerted a negative influence on general conditions at the time of the VFSS. The results of the current study show that dysphagia should be an early warning sign of disease progression and shorter survival in patients with PD.

This study had several limitations because patients with mild dysphagia who did not undergo VFSS were not included, pharyngeal dysmotility and silent aspiration in the early stages of pneumonia were not assessed. In addition, liquid swallows are usually used for assessing the timing of swallowing reflex; however, we did not use a liquid consistency, but rather a small amount of jelly consistency for the purposes of safety, tolerability, and preventing VFSS-related pneumonia [31]. Though many clinicians use liquid for VFSS adjusting the viscosity to prevent VFSS-related pneumonia patients with PD and dysphagia are prone to aspiration of liquid consistencies, without a cough response, potentially incurring VFSS-related aspiration pneumonia and clinical deterioration due to systemic inflammation [32]. If 3 mL of liquid had been used for VFSS, PDVFS might have been a different one. Therefore, PDVFS is suitable for the assessment of swallowing jelly and the validity of PDVFS using liquid should be verified in the future. Lastly, the patients enrolled were clinically diagnosed as exhibiting PD, however data collection was retrospective and single-center. In this study both prediction and validation were performed on the same group of patients. It is well known that predictive models derived from one population may have vastly different predictive validity when used in another population. This point is also a significant limitation. To validate the efficacy of PDVFS, further prospective multicenter studies are necessary.

## Conclusions

The VFSS features of mastication, lingual motility prior to transfer, aspiration, and total swallow time based on jelly swallowing were strongly associated with the development of aspiration pneumonia in patients with PD. Our newly-developed PDVFS could predict subsequent aspiration pneumonia and poor prognosis in such patients.

## Supporting information

S1 Fig ROC curve analysis and associated data of lingual pumping and four timed parameters. (PDF)

S2 Fig ROC curve analyzes of PDVFS stratified by (A) age (>73 or ≤73), (B) cognitive impairment (MMSE >24 or ≤24), (C) disease severity (Hoehn-Yahr stage (H-Y) 1–3 or 4–5), and (D) dietary interventions. (PDF)

S1 Table List of selected studies on the abnormal VFSS features in PD. (DOCX)

S2 Table Definitions of the 26 VFSS parameters. *Article No. was cited from S1 Table.
(DOCX)
