# Supplementary material for: Video-fluoroscopic swallowing study scale for predicting aspiration pneumonia in Parkinson’s disease
Source: PLoS One. 2018 Jun 6;13(6):e0197608. doi: 10.1371/journal.pone.0197608 (PMC5991364; doi:10.1371/journal.pone.0197608)
Supplement: S1 Fig — (PDF) [file pone.0197608.s001.pdf]

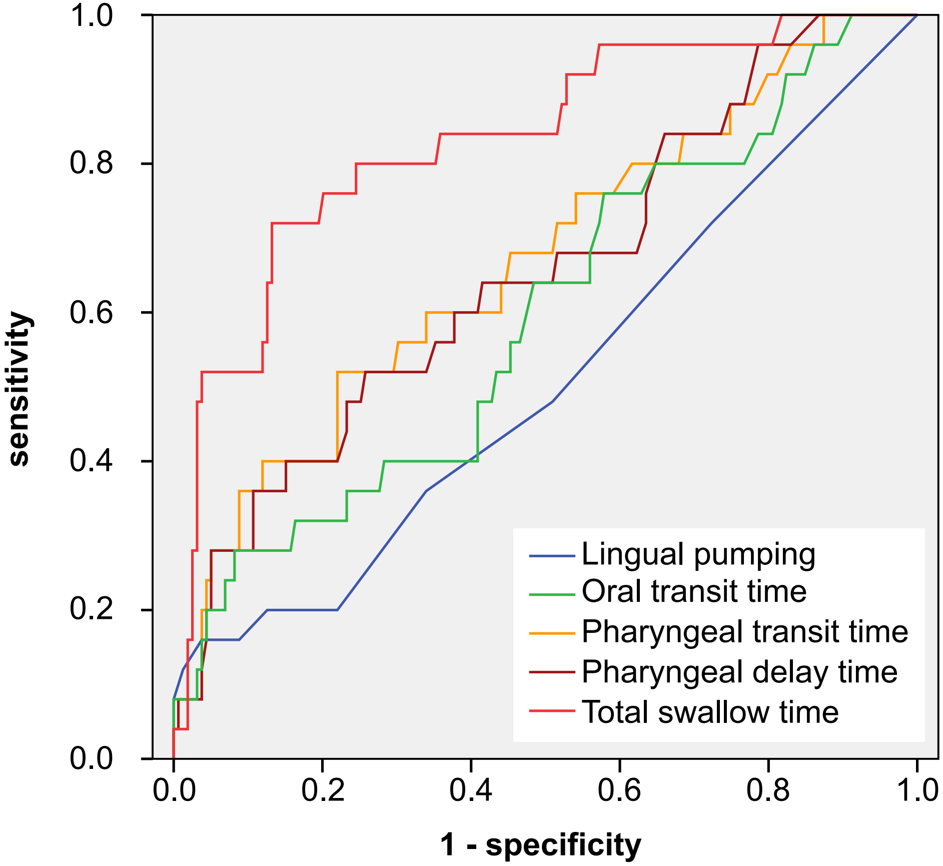

	AUC	Cut-off value	Sensitivity	Specificity
Lingual pumping	0.51	3	0.36	0.66
Oral transit time	0.60	5.0	0.28	0.91
Pharyngeal transit time	0.67	5.0	0.36	0.91
Pharyngeal delay time	0.65	4.0	0.36	0.89
Total swallow time	0.83	10.0	0.72	0.85
