# Supplementary figures and images for: Video-fluoroscopic swallowing study scale for predicting aspiration pneumonia in Parkinson’s disease
Source: PLoS One. 2018 Jun 6;13(6):e0197608. doi: 10.1371/journal.pone.0197608 (PMC5991364; doi:10.1371/journal.pone.0197608)

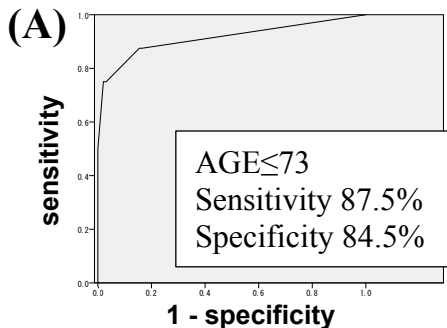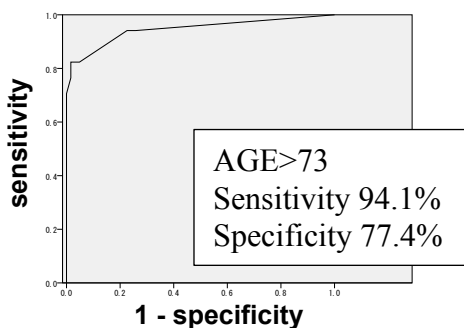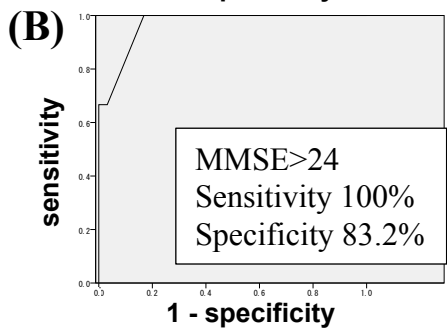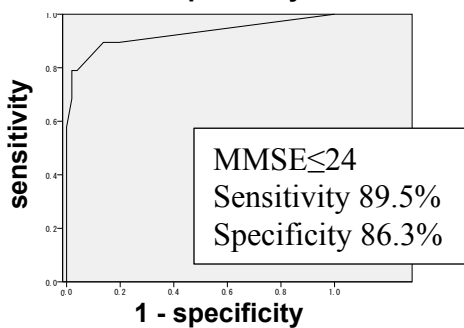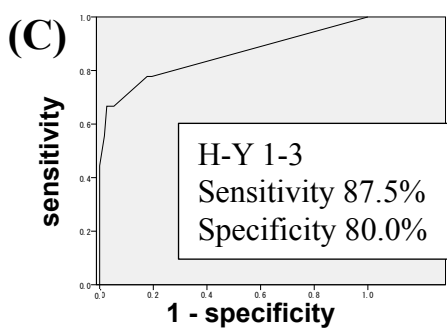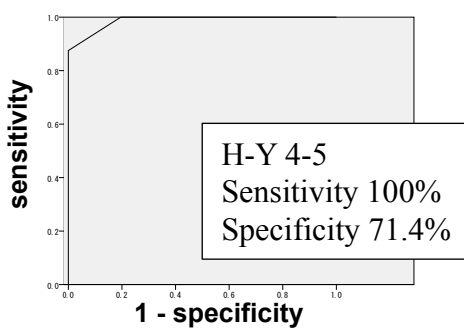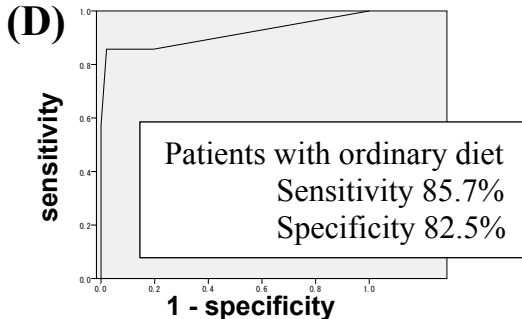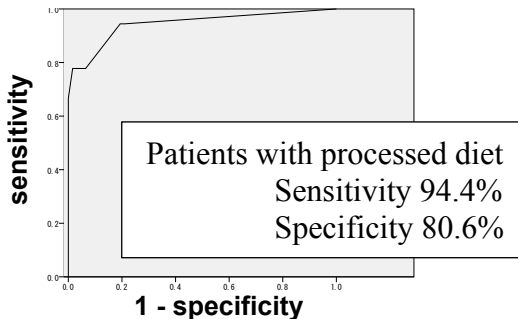

Supplement: S2 Fig — (PDF) [file pone.0197608.s002.pdf]
