# Supplementary material for: Video-fluoroscopic swallowing study scale for predicting aspiration pneumonia in Parkinson’s disease
Source: PLoS One. 2018 Jun 6;13(6):e0197608. doi: 10.1371/journal.pone.0197608 (PMC5991364; doi:10.1371/journal.pone.0197608)
Supplement: S1 Table — (DOCX) [file pone.0197608.s003.docx]

**S1 Table List of selected studies on the abnormal VFSS features in PD**

| **Article No.** | **Published articles (Author, Journal name, publication year, page)** |
| --- | --- |
| 1 | Robbins JA, et al. Ann Neurol. 1986;19(3):283-7. |
| 2 | Feinberg MJ, et al. AJR Am J Roentgenol. 1991;156(2):293-6. |
| 3 | Bird MR, et al. Age Ageing. 1994;23(3):251-4. |
| 4 | Leopold NA, et al. Dysphagia. 1996;11(1):14-22. |
| 5 | Leopold NA, et al. Dysphagia. 1997;12(1):11-8; discussion 9-20. |
| 6 | Johnston BT, et al. Mov Disord. 1997;12(3):322-7. |
| 7 | Nagaya M, et al. Dysphagia. 1998;13(2):95-100. |
| 8 | O'Neil KH, et al. 1999;14(3):139-45. |
| 9 | Volonte MA, et al. Neurol Sci. 2002;23 Suppl 2:S121-2. |
| 10 | Pfeiffer RF, et al. Lancet Neurol. 2003;2(2):107-16. |
| 11 | Potulska A, et al. Parkinsonism Relat Disord. 2003;9(6):349-53. |
| 12 | Monte FS, et al. Mov Disord. 2005;20(4):457-62. |
| 13 | Troche MS, et al. Dysphagia. 2008;23(1):26-32. |
| 14 | Cappabianca S, et al. Radiol Med. 2008;113(6):923-40. |
| 15 | Menezes C, et al. J Clin Pharm Ther. 2009;34(6):673-6. |
| 16 | Yamamoto T, et al. Parkinsonism Relat Disord. 2010;16(8):503-6. |
| 17 | Baijens LW, et al. Gastroenterol Res Pract. 2011;2011:380682 |
| 18 | Umemoto G, et al. Dysphagia. 2011;26(3):250-5. |
| 19 | Lin CW, et al. Arch Phys Med Rehabil. 2012;93(11):2080-4. |
| 20 | Londos E, et al. BMC Neurol. 2013;13:140. |
| 21 | Kim J, et al. Dysphagia. 2014;29(4):438-43. |
| 22 | Argolo N, et al. Int J Lang Commun Disord. 2015;50(5):659-64. |
| 23 | Kim YH, et al. Laryngoscope. 2015;125(2):389-95. |
| 24 | Rajaei A, et al. Adv Biomed Res. 2015;4:108. |
