# Supplementary material for: Video-fluoroscopic swallowing study scale for predicting aspiration pneumonia in Parkinson’s disease
Source: PLoS One. 2018 Jun 6;13(6):e0197608. doi: 10.1371/journal.pone.0197608 (PMC5991364; doi:10.1371/journal.pone.0197608)
Supplement: S2 Table — *Article No. was cited from S1 Table. (DOCX) [file pone.0197608.s004.docx]

**S2 Table. Definitions of the 26 VFSS parameters**

| Parameter | Definitions | Coded value | Article No.^*^ |
| --- | --- | --- | --- |
| **Oral phase** |  |  |  |
| Pre-swallow anterior spill | Pre-swallow loss of bolus from the lips | 0: absent, 1: present | 17 |
| Lingual pumping | Number of times the tongue pumps (rocks) while the bolus is in the oral cavity, resulting in posterior movement of the bolus and initiation of the swallow reflex | 0: 1–3 times, 1: ≥4 times | 7, 13, 22 |
| Poor velopharyngeal closure | Failure of the velopharyngeal closure mechanism causes nasal regurgitation | 0: absent, 1: present | 8 |
| Swallow hesitancy | Difficulty initiating swallowing, mainly trouble with bolus transfer | 0: absent, 1: present | 17 |
| Piecemeal deglutition | Sequential swallowing of the bolus, which is fractionated into many swallowing units | 0: absent, 1: present | 1, 7, 10, 17, 18, 21, 22 |
| Lip closure | Maintaining lip closure throughout the oral phase | 0: intact, 1: inadequate | 8, 9, 21 |
| Mastication | Mastication is slow, hesitant, and delayed with ineffectual movements | 0: intact, 1: inadequate | 4, 6, 9, 21 |
| Lingual motility prior to transfer | Tongue movement assisting mastication and bolus formation | 0: intact, 1: inadequate | 4 |
| Bolus formation | Creating a cohesive bolus by mastication prior to the remaining phases of the swallow | 0: intact, 1: inadequate | 8, 10, 14, 20, 21 |
| Premature bolus loss | Posterior bolus leakage prior to active transfer; because of impaired oral containment | 0: absent, 1: present | 2, 7, 21, |
| Palatal elevation | The tongue presses against the palate to transfer the bolus and prevent spillage | 0: intact, 1: inadequate | 9, 14, 21, |
| Poor bolus propulsion | Latent, uncoordinated, premature, and segmented lingual transfers of bolus | 0: absent, 1: present | 8 |
| Residue in oral cavity | Oral retention of bolus | 0: absent to mild,  1: moderate to severe | 7, 10, 12, 17, 21 |
| **Pharyngeal phase** |  |  |  |
| Triggering of pharyngeal swallow | Delayed onset of the pharyngeal phase of swallow | 0: intact, 1: delayed | 9, 11, 21 |
| Vallecular residue | Retention of bolus in one or both vallecular spaces | 0: absent, 1: present | 3, 5, 6, 7, 10, 17, 21 |
| Laryngeal elevation | Insufficient movement or delayed onset of laryngeal elevation | 0: intact, 1: inadequate | 5, 7, 8, 21 |
| Pyriform sinus residue | Retention of bolus in one or both pyriform sinus | 0: absent, 1: present | 5, 6, 7, 8, 12, 17, 20, 21 |
| Reduced epiglottal tilt | Decreased epiglottic range of motion during the pharyngeal phase of swallow | 0: intact, 1: inadequate | 2, 8, |
| Coating of pharyngeal wall | Retention of bolus along the pharyngeal wall | 0: absent, 1: present | 14, 21 |
| Repeated swallowing | Multiple swallows during the pharyngeal phase | 0: absent, 1: present | 14, 21 |
| Aspiration | Entry of bolus into the lower respiratory tract | 0: absent, 1: present | 1, 2, 3, 5, 8, 12, 16, 17, 20, 21, 23, 24 |
| Cricopharyngeal dysfunction | Impaired relaxation of the cricopharyngeal muscle (referred to as cricopharyngeal spasm or cricopharyngeal achalasia) | 0: absent, 1: present | 2, 8, 9, 10 |
| **Timed parameters** |  |  |  |
| Oral transit time | From onset of posterior movement by the bolus in the oral cavity, and point at which the tongue tip is raised and the bolus begins posterior movement toward the posterior aspect of the oral cavity to the point at which the tail of the bolus passes the level of the ramus of the mandible | 0: ≤5 sec, 1: >5 sec | 3, 7, 12, 15, 18, 19, 21, 23 |
| Pharyngeal transit time | From a point at which the tail of the bolus passes the level of the ramus of the mandible to the point at which the bolus passes through the upper esophageal sphincter | 0: ≤5 sec, 1: >5 sec | 15, 18, 19, 21, 23 |
| Pharyngeal delay time | From the arrival of the bolus head at the point where the shadow of the lower edge of the mandible crosses the tongue base until laryngeal elevation, indicating the onset of the pharyngeal swallow | 0: ≤4 sec, 1: >4 sec | 23 |
| Total swallow time | From the initiation of mastication until the tail of the bolus passed through the upper esophageal sphincter | 0: ≤10 sec, 1: >10 sec | 23 |
